# Hydrothermal plume detection dataset from Chinese cruises to the equatorial East Pacific Rise

**DOI:** 10.1016/j.dib.2020.106540

**Published:** 2020-11-20

**Authors:** Sheng Chen, Chunhui Tao, Christopher R. German

**Affiliations:** a Ocean Technology and Equipment Research Center, School of Mechanical Engineering, Hangzhou Dianzi University, Hangzhou 310018, China; b Department of Geology and Geophysics, Woods Hole Oceanographic Institution, Woods Hole, MA 02543, United States; c Key Laboratory of Submarine Geosciences, SOA & Second Institute of Oceanography, MNR, Hangzhou 310012, China; d School of Oceanography, Shanghai Jiao Tong University, Shanghai 200240, China

**Keywords:** Hydrothermal detection, MAPR, Turbidity, ORP, Low temperature, East Pacific Rise

## Abstract

In this data article, a dataset from hydrothermal plume investigations on East Pacific Rise collected during Chinese cruises from 2008 to 2011 is reported. The dataset is related to the research article entitled “Abundance of low-temperature axial venting at the equatorial East Pacific Rise” published in the journal Deep-Sea Research I by Chen et al. (2020). In the dataset, continuous strings of time-series sensor data were obtained by Miniature Autonomous Plume Recorders (MAPR) and an Oxidation-Reduction Potential (ORP) sensor, while the underwater position data was derived using Ultra Short Base Line (USBL) navigation. In this contribution, general characteristics of the data are summarized and showed here. All the data are stored in separate Microsoft Excel spreadsheets that are available for researchers and a link is provided to the full data at http://dx.doi.org/10.17632/jckyj5vyjx.1. The data will be of comparative value to those investigating hydrothermal activities along mid-ocean ridges, worldwide.

## Specifications Table

**Table:** 

Subject	Marine Geology
Specific subject area	Hydrothermal activity
Type of data	Tables and Microsoft Excel
How data were acquired	Turbidity data was collected by MAPR sensors. ORP data was obtained by ORP sensor. Underwater position data was derived from USBL.
Data format	Raw
Parameters for data collection	Fieldwork for this dataset was stimulated by initial indications of hydrothermal activity along the EPR detected during Chinese cruise DY115–17.
Description of data collection	All the data were collected during Chinese research cruises between 2008 and 2011.
Data source location	Key Laboratory of Submarine Geosciences, SOA & Second Institute of Oceanography, MNR, Hangzhou, 310,012, China.
Data accessibility	http://dx.doi.org/10.17632/jckyj5vyjx.1
Related research article	Sheng Chen, Chunhui Tao, Christopher R. German, Abundance of low-temperature axial venting at the equatorial East Pacific Rise. Deep-Sea Research I. DOI:http://dx.doi.org/10.1016/j.dsr.2020.103426

## Value of the Data

• These data on hydrothermal plume distributions are from an understudied portion of the East Pacific Rise (1.9°N to 4.9°S) collected over multiple expeditions. Making these data available will facilitate comparison with other hydrothermal data-sets, worldwide.
• The data will be of value to those working on hydrothermal activity, particularly when considering heat flow, water volume fluxes, mineralization and biogeochemical cycles.
• This dataset can be used in further research pursuing data synthesis and/or regional comparisons on multiple spatial and/or temporal scales.
• These data fill a gap for the equatorial EPR in the international InterRidge data-base and provide the potential to investigate the importance of low temperature axial venting to geophysical fluxes and geochemical equilibrium.

## 1 Data Description

This Data in Brief article provides figures and data sets of hydrothermal plume investigations of the equatorial East Pacific Rise (EPR), collected during Chinese research cruises between 2008 and 2011.

**Fig. 1:**
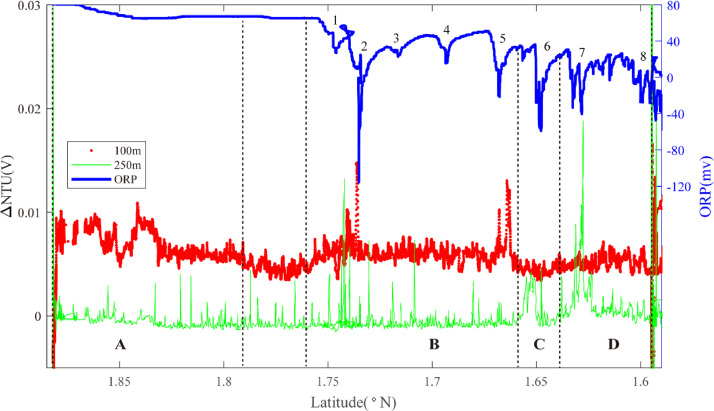
The plot of turbidity and ORP anomaly of Line 01 (22VIII-L06) on segment 1. Letters A-D and numbers 1–8 refer to the hydrothermal anomaly regions discussed in the text.

**Fig. 2:**
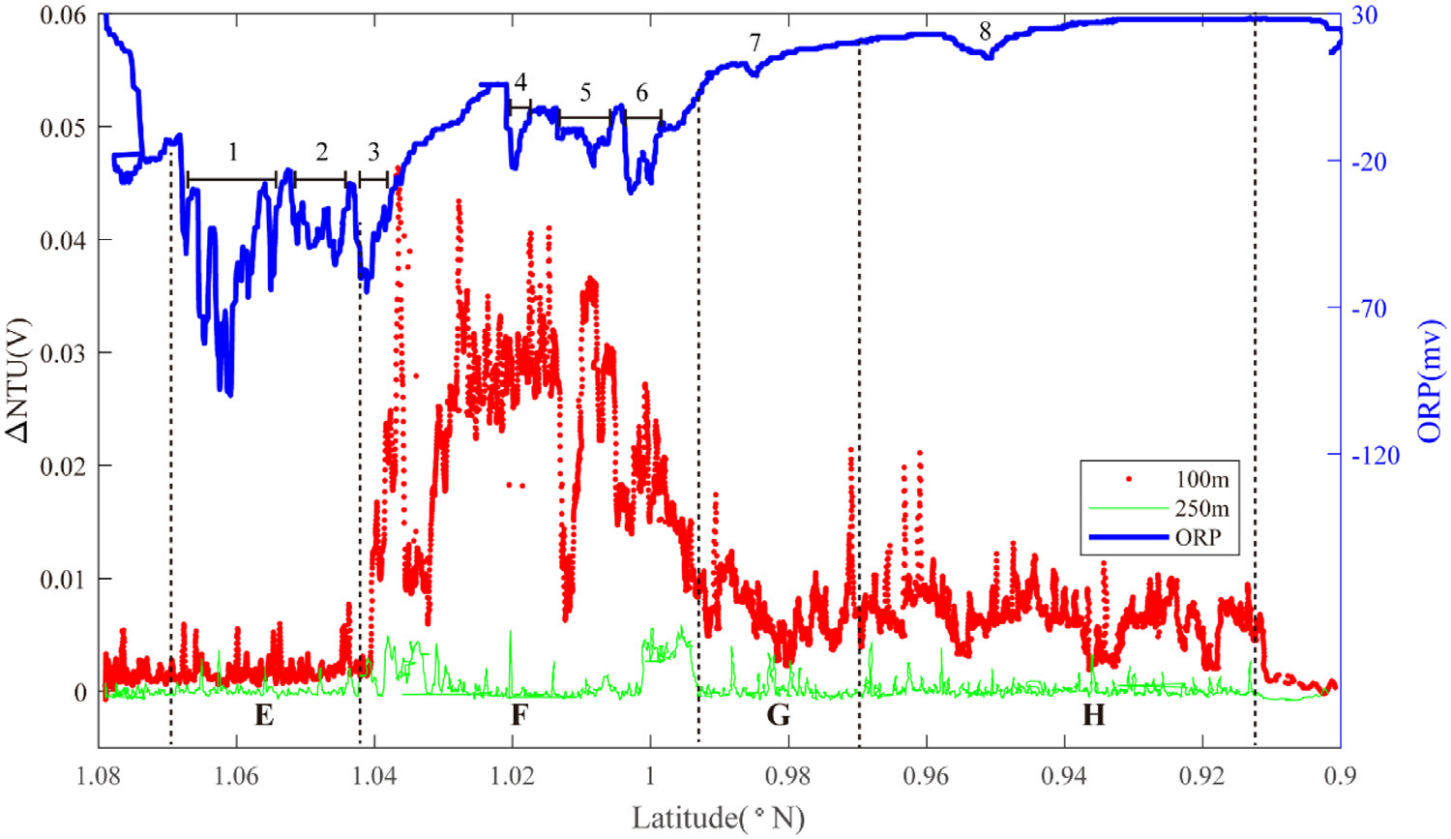
The plot of turbidity and ORP anomaly of Line 04 (22VIII-L03) on segment 2–1. Letters E-H and numbers 1–8 refer to the hydrothermal anomaly regions discussed in the text.

**Fig. 3:**
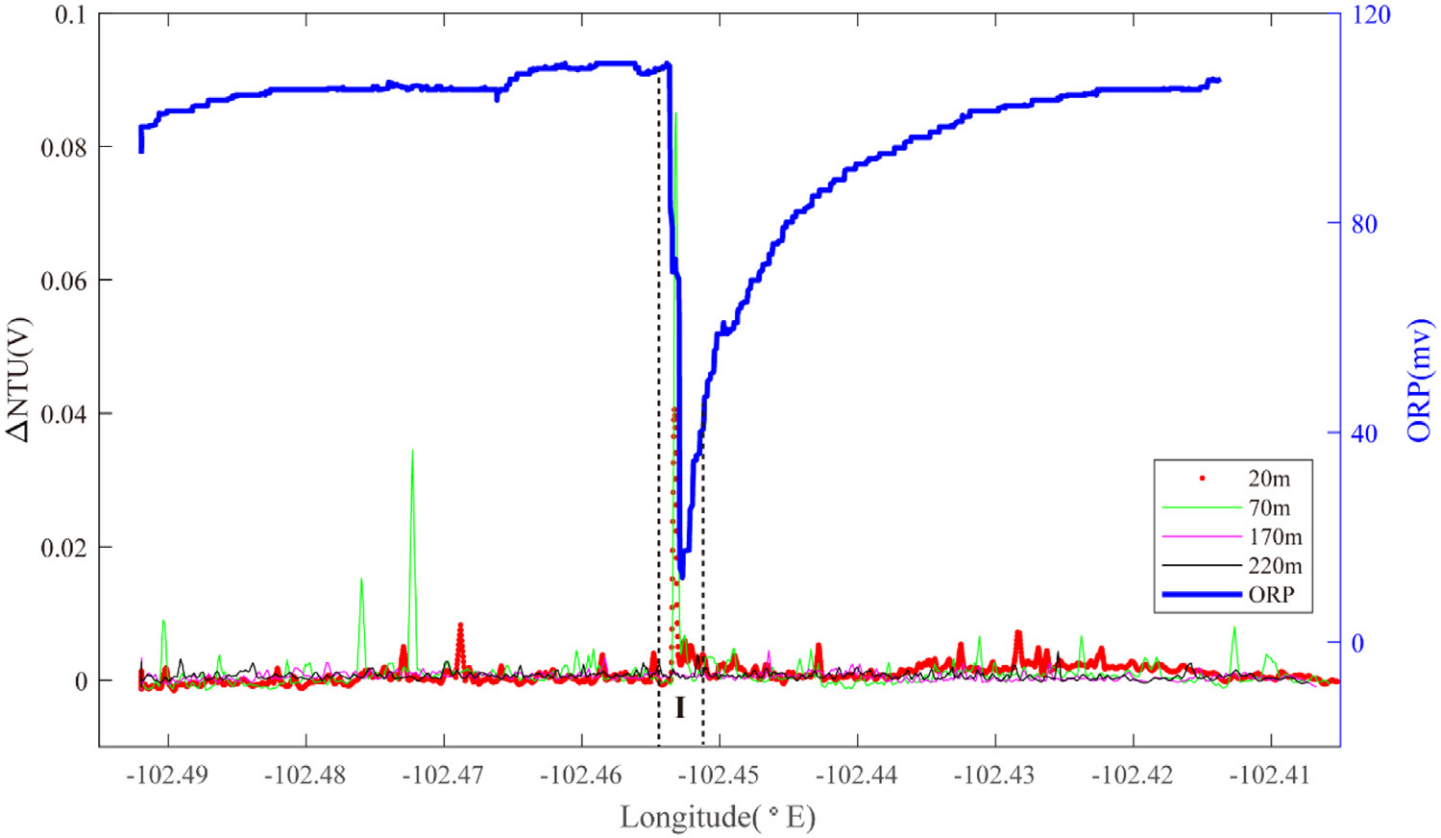
The plot of turbidity and ORP anomaly of Line 06 (20III-L06) on segment 2–3. Letter I refers to the hydrothermal anomaly region discussed in the text.

**Fig. 4:**
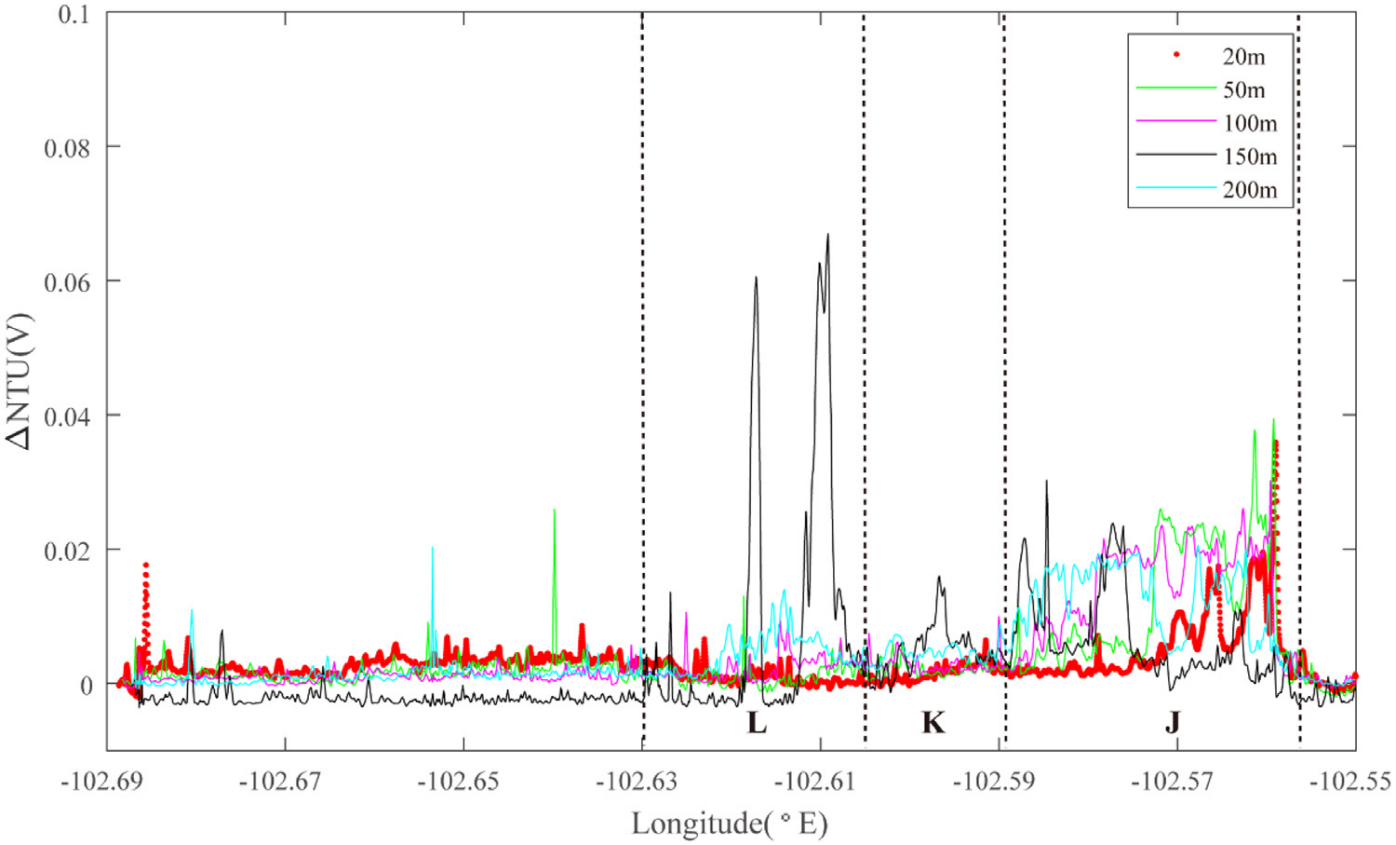
The plot of turbidity anomaly of Line 07 (20III-L09) on segment 2–4. Letters J-L refer to the hydrothermal anomaly regions discussed in the text.

**Fig. 5:**
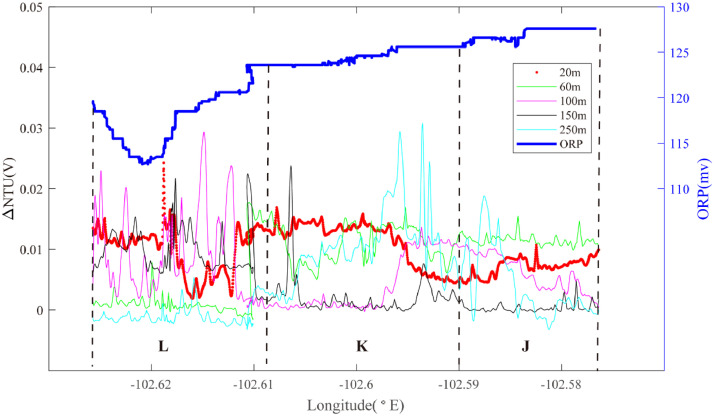
The plot of turbidity and ORP anomaly of Line 08 (20III-L13) on segment 2–4. Letters J-L refer to the hydrothermal anomaly regions discussed in the text.

**Fig. 6:**
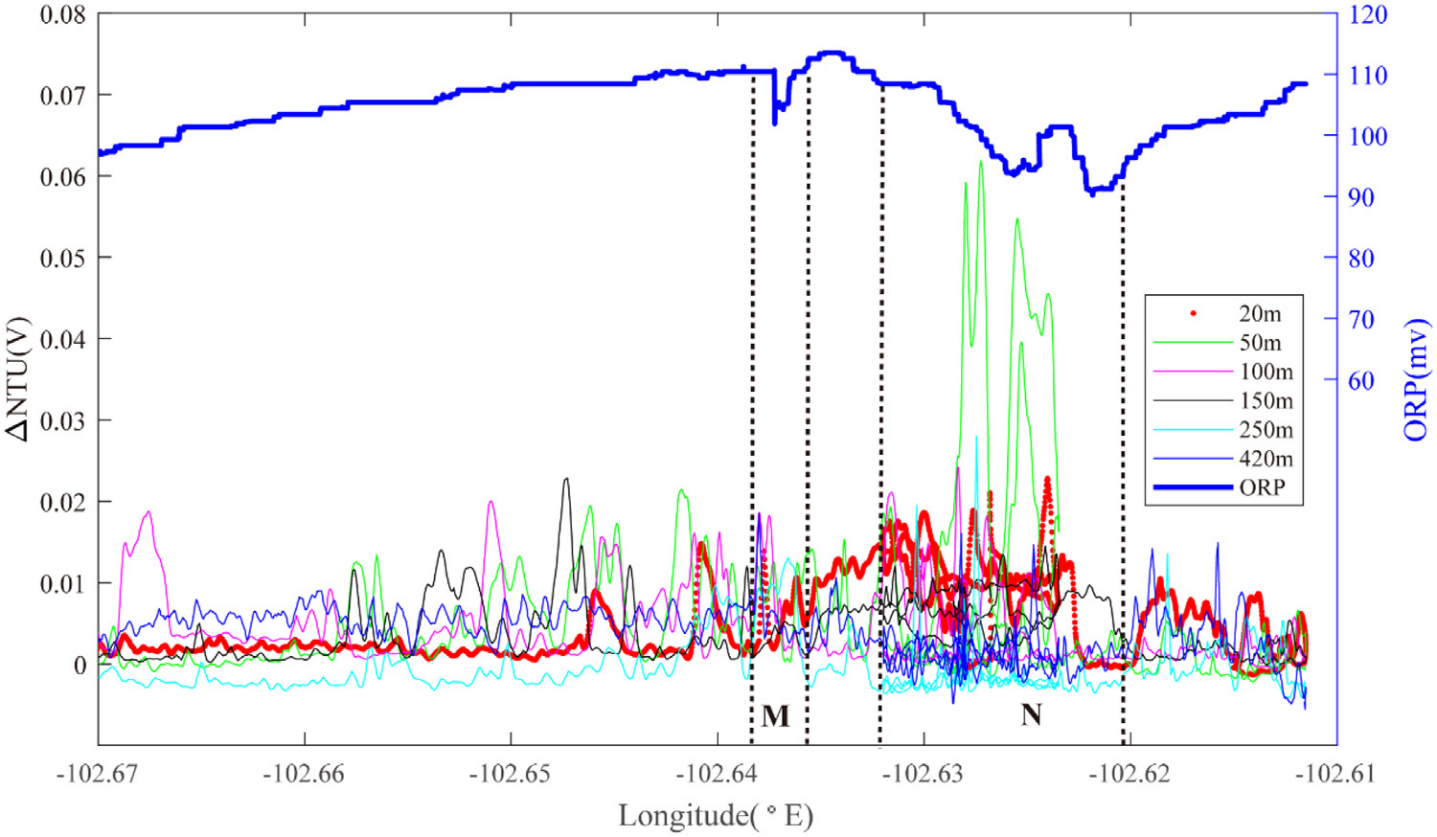
The plot of turbidity and ORP anomaly of Line 09 (20III-L12) on segment 2–4. Letters M & N refer to the hydrothermal anomaly regions discussed in the text.

**Fig. 7:**
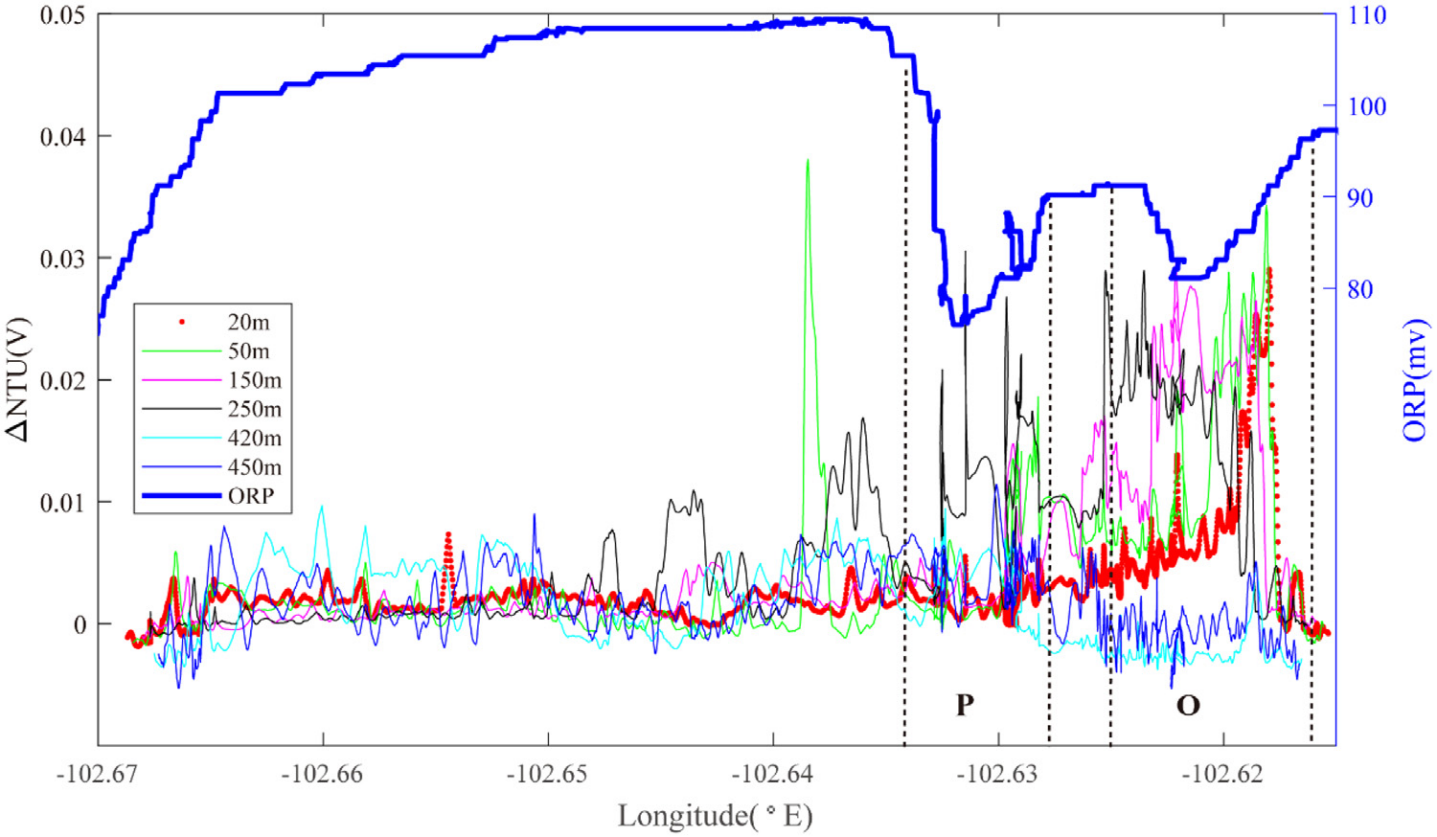
The plot of turbidity and ORP anomaly of Line 10 (20III-L11) on segment 2–4. Letters O&P refer to the hydrothermal anomaly regions discussed in the text.

**Fig. 8:**
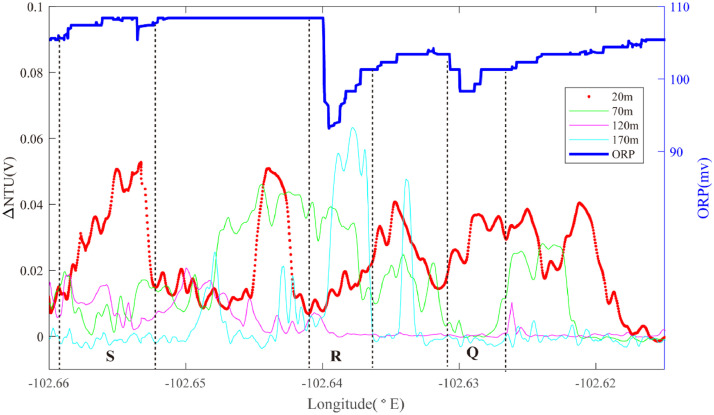
The plot of turbidity and ORP anomaly of Line 12 (20III-L07b) on segment 2–4. Letters Q-S refer to the hydrothermal anomaly regions discussed in the text.

**Fig. 9:**
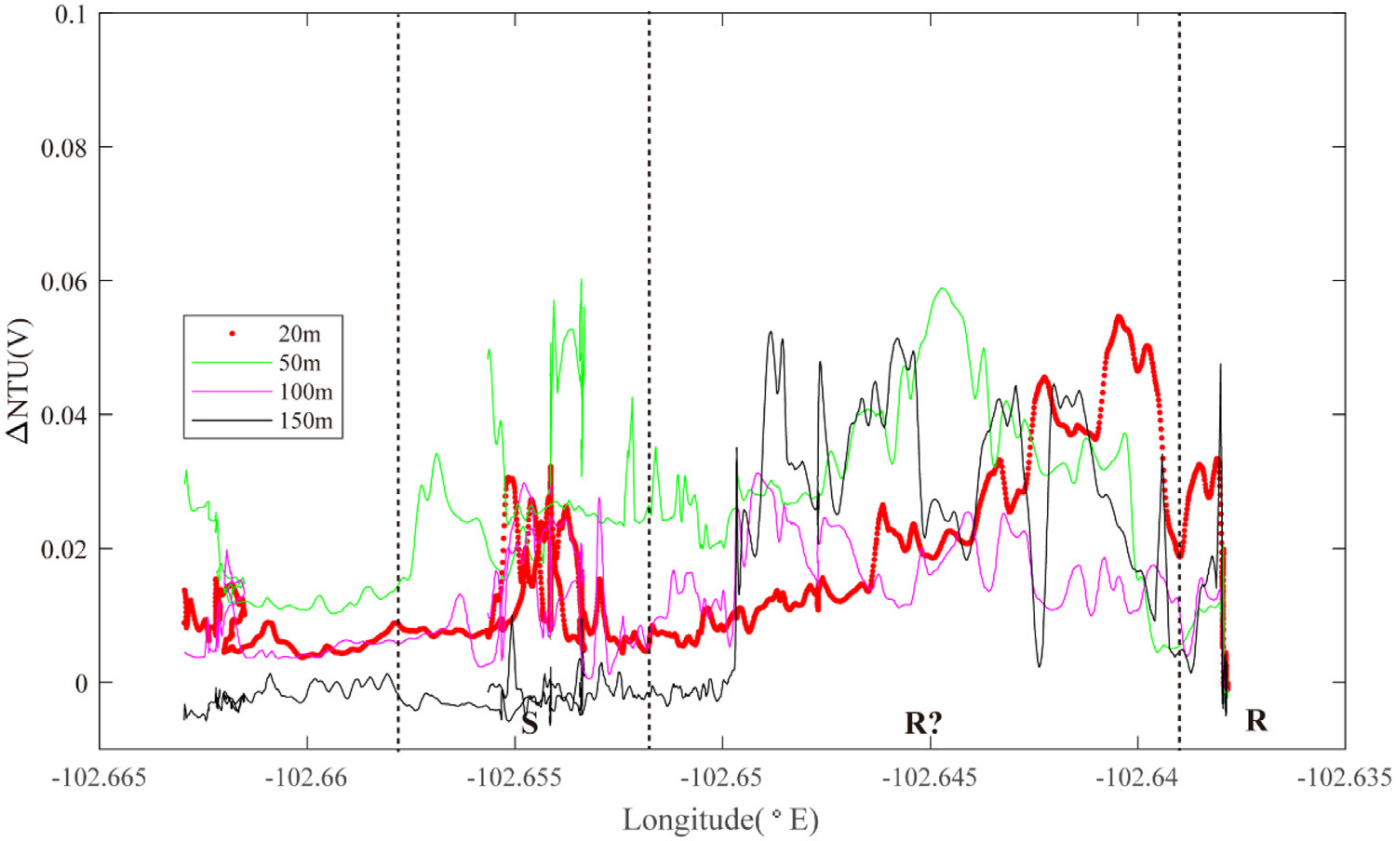
The plot of turbidity and ORP anomaly of Line 13 (20III-L08ab) on segment 2–4. Letter R refers to the hydrothermal anomaly region discussed in the text.

**Fig. 10:**
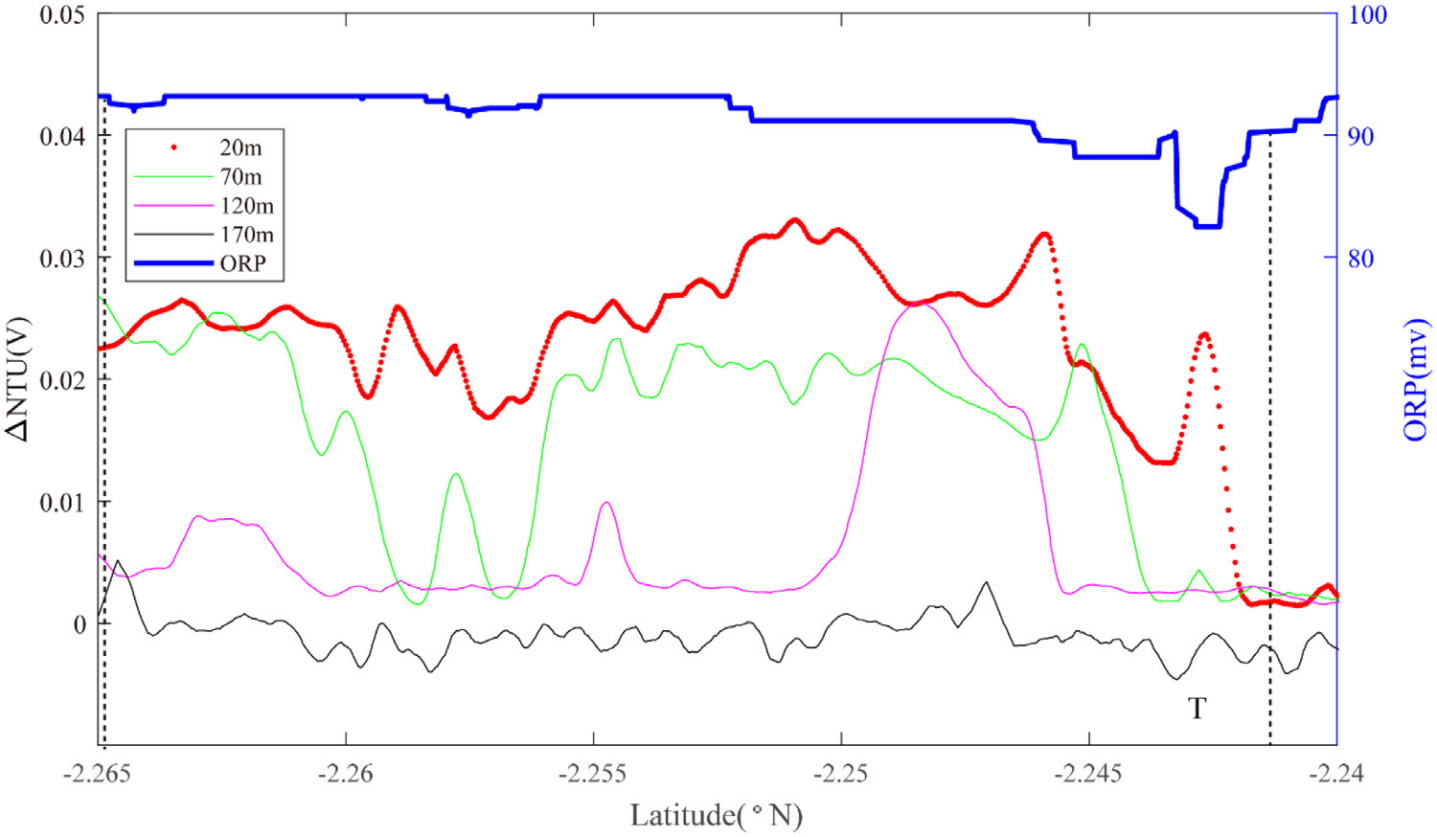
The plot of turbidity and ORP anomaly of Line 11 (20III-L07a) on segment 2–4. Letter T refers to the hydrothermal anomaly region discussed in the text.

**Fig. 11:**
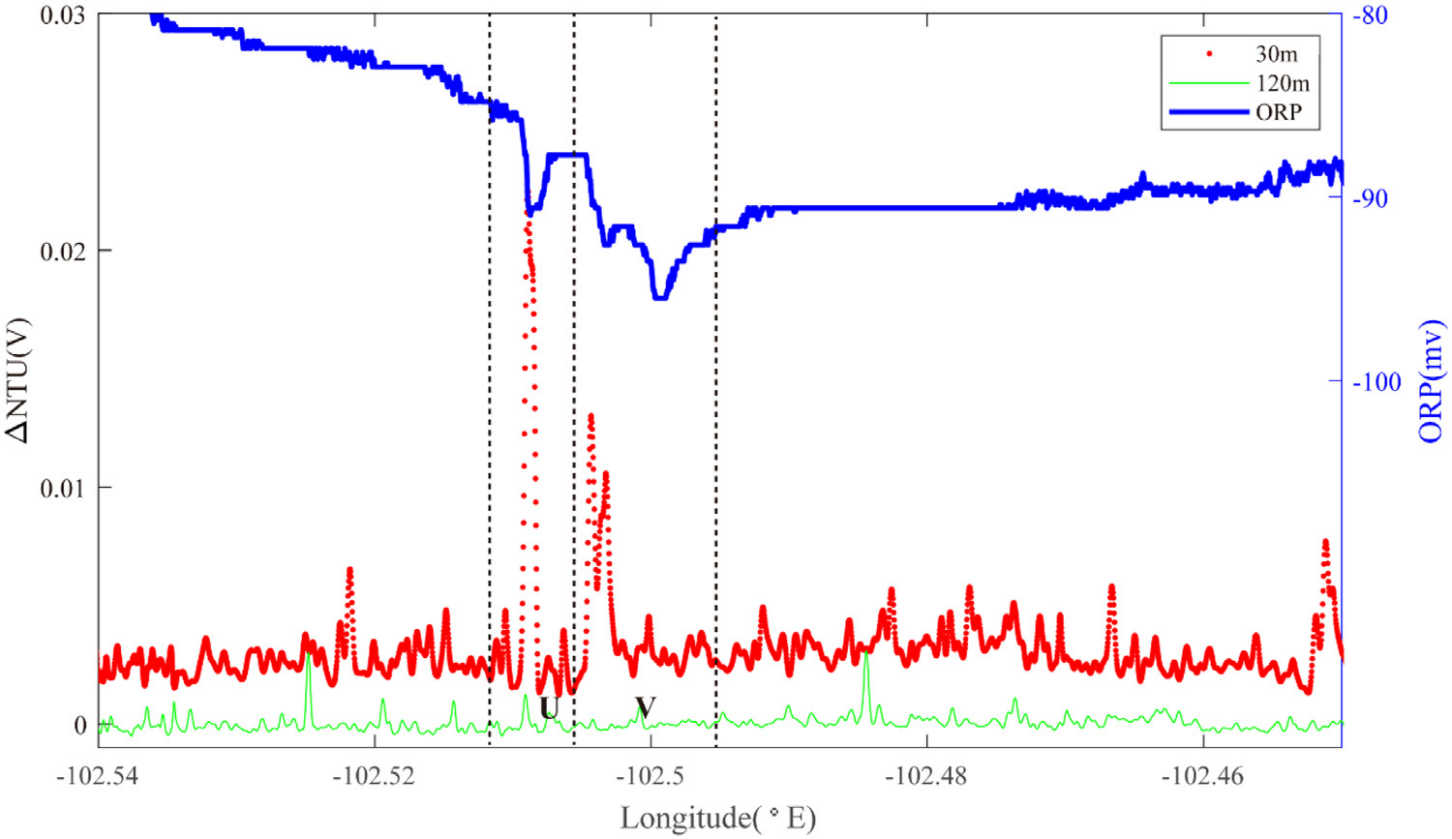
The plot of turbidity and ORP anomaly of Line 15(22VI-L09) on segment 2–5. Letters U & V refer to the hydrothermal anomaly regions discussed in the text.

**Fig. 12:**
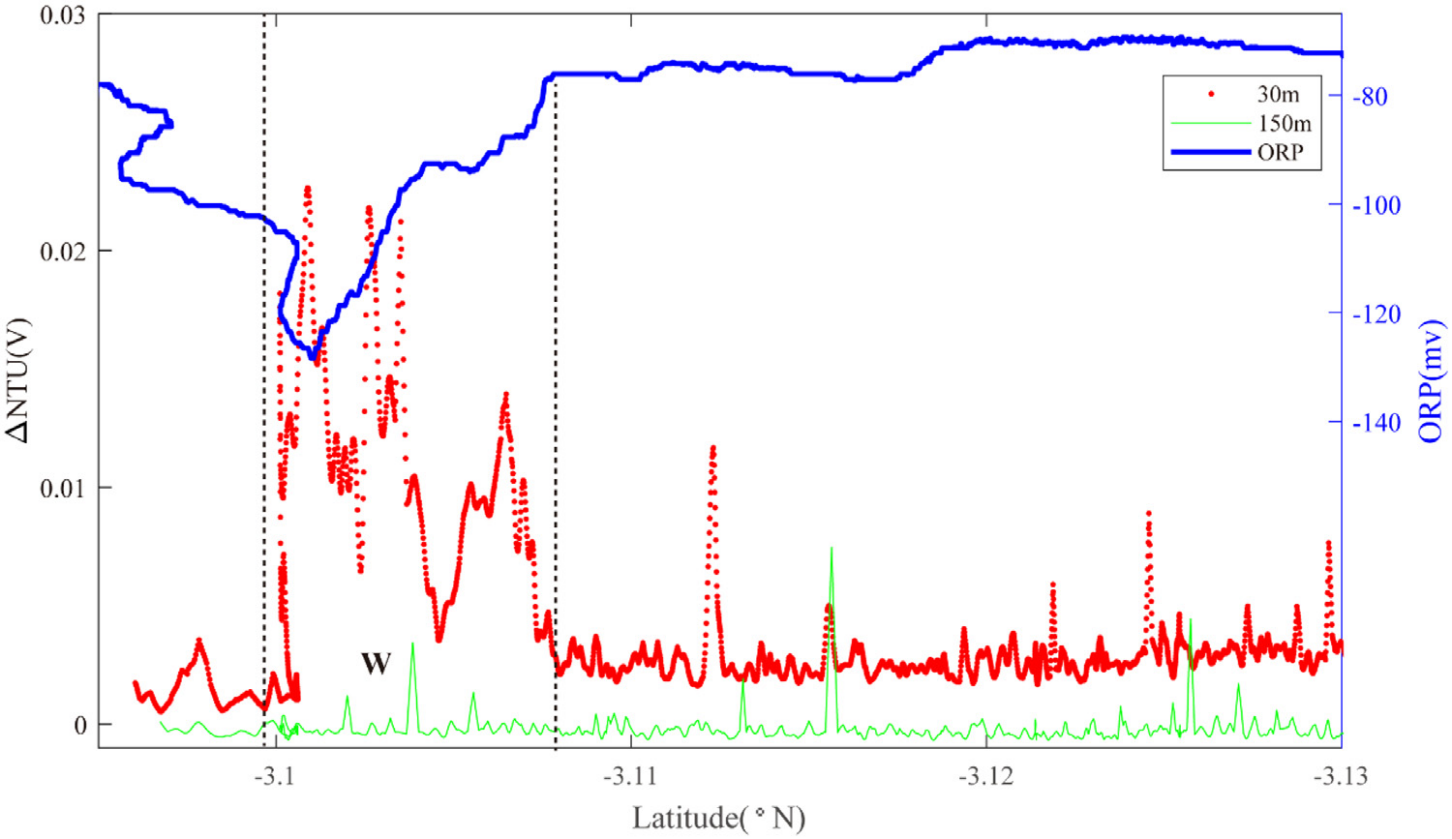
The plot of turbidity and ORP anomaly of Line 18(22VI-L07) on segment 2–5. Letter W refers to the hydrothermal anomaly region discussed in the text.

**Fig. 13:**
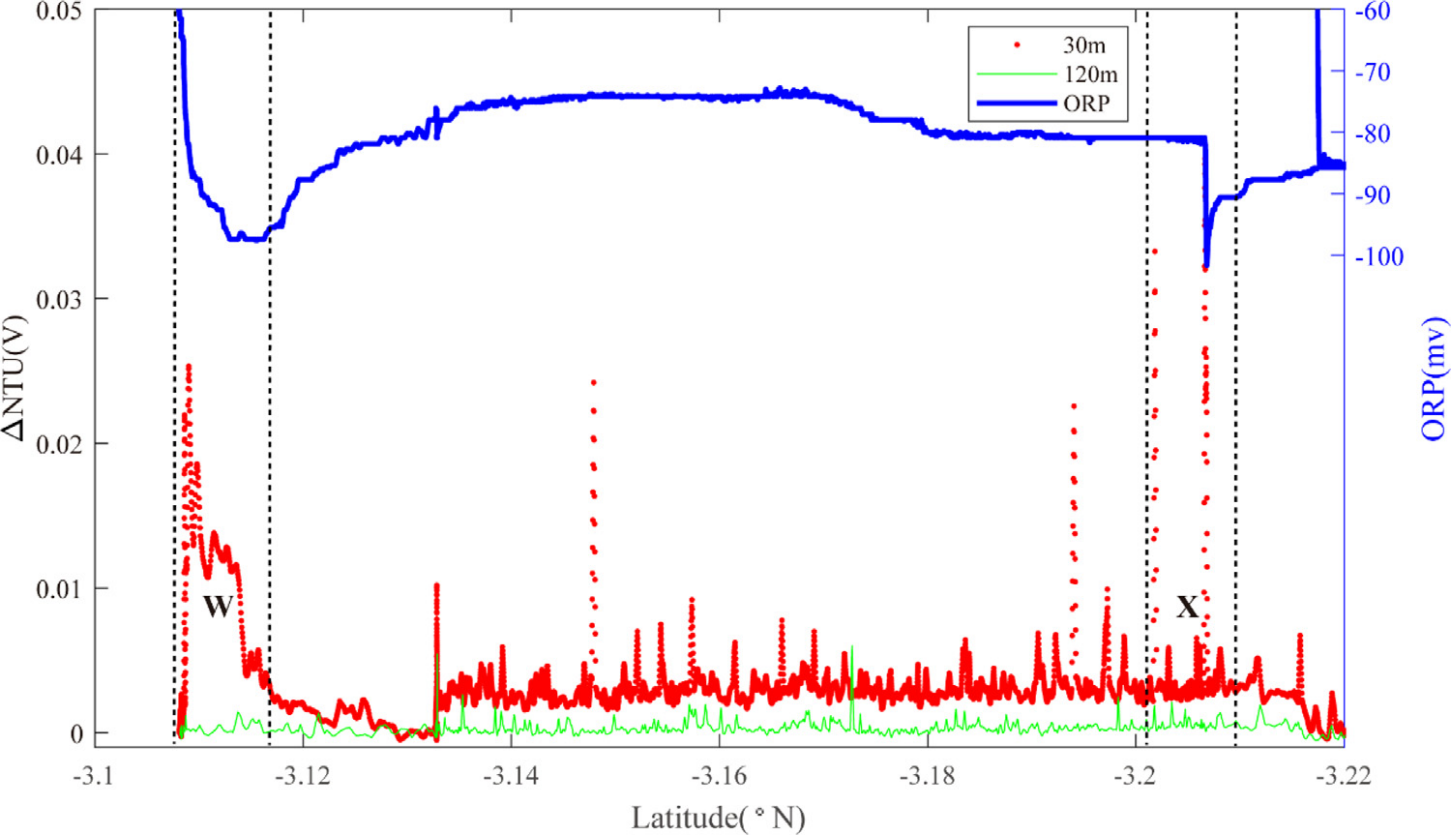
The plot of turbidity and ORP anomaly of Line 19(22VI-L08) on segment 2–5. Letters X&W refers to the hydrothermal anomaly region discussed in the text.

**Fig. 14:**
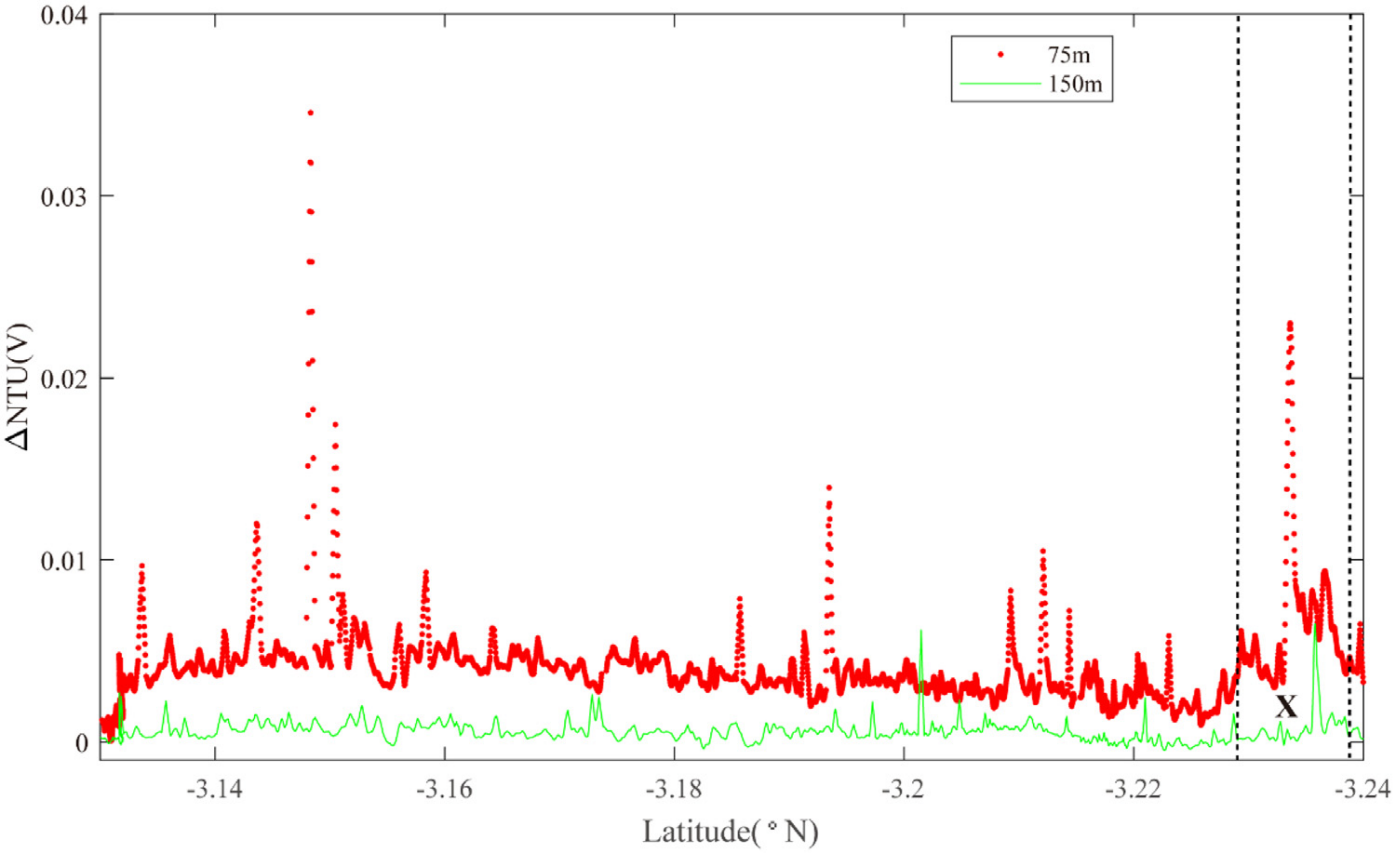
The plot of turbidity anomaly of Line 17(22VI-L06) on segment 2–5. Letter X refers to hydrothermal anomaly discussed in the text.

**Fig. 15:**
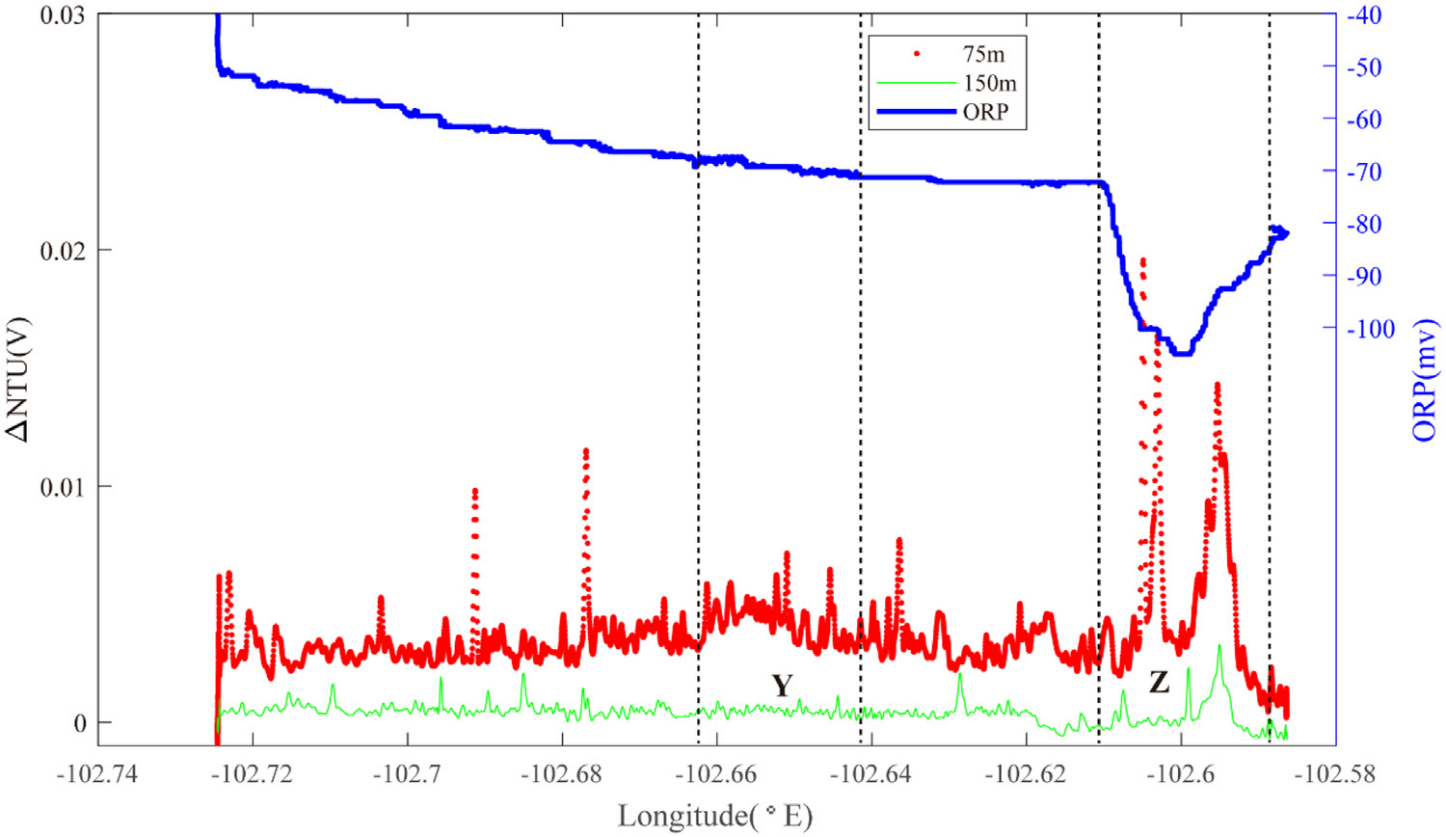
The plot of turbidity and ORP anomaly of Line 20 (22VI-L03) on segment 2–5. Letters Y&Z refers to hydrothermal anomaly discussed in the text.

Hydrothermal plume sensor data are presented for 26 deep-tow survey lines (Table 1), including MAPR (Miniature Autonomous Plume Recorders) data, ORP (Oxidation-Reduction Potential) data and underwater position data, which can all be downloaded at http://dx.doi.org/10.17632/jckyj5vyjx.1. The data for each line are placed together in a common folder. All survey lines are grouped together in separate folders associated with each of the ridge segments studied, such as “Segment 1″, “Segment 2–1″. All the data are stored in separate Microsoft Excel spreadsheets. Each survey line is briefly summarized in Table 1, including organizational names initially assigned during the research cruises, starting longitude, starting latitude, end longitude, end latitude, line name in the associated research article, starting time and end time. Table 2 provides calculations of the effective survey lengths of each line listed in Table 1. The track lines for each of these surveys are also published in map form in “Fig. 1” of the associated research publication by Chen et al. (2020) [1] with color coding (red, black) to indicate which of the surveys did or did not reveal evidence of seafloor fluid flow, respectively. Here, Fig. 1, Fig. 2, Fig. 3, Fig. 4, Fig. 5, Fig. 6, Fig. 7, Fig. 8, Fig. 9, Fig. 10, Fig. 11, Fig. 12, Fig. 13, Fig. 14, Fig. 15 present plots of optical sensor and redox sensor responses along the track line (amplitude vs distance). These are the primary data used for the analysis presented in Chen et al. (2020) [1].

**Table 1:** Catalog of all survey track lines reported.

Seg	Locations	Organised_Name	Start_Lat	Start_Lon	End_Lat	End_Lon	Line_Name	Start_Time	End_Time
S1	1.9°N-1.2°N	Line 01	1.88	−102.28	1.59	−102.26	22VIII-L06	2011/10/04 06:20	2011/10/05 02:58
S2–1	1.2°N-0.7°N	Line 02	1.17	−102.27	1.07	−102.28	22VIII-L01	2011/09/22 16:20	2011/09/23 02:14
		Line 03	1.18	−102.16	1.08	−102.18	22VIII-L02	2011/09/23 05:00	2011/09/23 17:07
		Line 04	1.08	−102.18	0.90	−102.21	22VIII-L03	2011/09/24 11:00	2011/09/25 05:14
S2–3	1.2°S-1.5°S	Line 05	−1.37	−102.50	−1.35	−102.50	20III-L05	2008/08/22 10:30	2008/08/22 16:14
		Line 06	−1.36	−102.48	−1.39	−102.41	20III-L06	2008/08/22 20:30	2008/08/23 08:03
S2–4	1.5°S-2.8°S	Line 07	−2.02	−102.68	−2.01	−102.56	20III-L09	2008/08/25 05:00	2008/08/25 18:49
		Line 08	−2.02	−102.62	−2.02	−102.58	20III-L13	2008/08/28 09:45	2008/08/28 19:08
		Line 09	−2.05	−102.67	−2.09	−102.61	20III-L12	2008/08/27 22:20	2008/08/28 09:19
		Line 10	−2.17	−102.67	−2.13	−102.62	20III-L11	2008/08/26 05:54	2008/08/26 11:31
		Line 11	−2.27	−102.66	−2.20	−102.59	20III-L07a	2008/08/23 23:15	2008/08/24 03:04
		Line 12	−2.27	−102.66	−2.20	−102.59	20III-L07b	2008/08/24 03:05	2008/08/24 08:57
		Line 13	−2.22	−102.66	−2.23	−102.64	20III-L08	2008/08/24 11:30	2008/08/25 02:29
		Line 14	−2.51	−102.71	−2.69	−102.62	22VI-L10	2011/07/17 18:00	2011/07/18 20:11
S2–5	2.8°S-4.0°S	Line 15	−2.93	−102.55	−3.03	−102.45	22VI-L09	2011/07/16 22:17	2011/07/17 11:16
		Line 16	−3.03	−102.55	−3.33	−102.64	22VI-L04&L05	2011/07/14 01:45	2011/07/14 16:56
		Line 17	−3.13	−102.60	−3.24	−102.55	22VI-L06	2011/07/14 18:12	2011/07/15 04:11
		Line 18	−3.13	−102.56	−3.10	−102.55	22VI-L07	2011/07/15 18:12	2011/07/16 06:28
		Line 19	−3.12	−102.56	−3.21	−102.58	22VI-L08	2011/07/16 06:30	2011/07/16 20:17
		Line 20	−3.60	−102.72	−3.64	−102.60	22VI-L03	2011/07/13 06:30	2011/07/13 18:30
		Line 21	−3.09	−102.57	−3.11	−102.54	22VI-L18	2011/07/24 09:45	2011/07/24 16:43
		Line 22	−3.11	−102.58	−3.11	−102.52	22VI-L19	2011/07/24 17:00	2011/07/25 00:18
		Line 23	−3.11	−102.57	−3.12	−102.54	22VI-L21	2011/07/27 20:00	2011/07/28 02:40
S3	3.7°S-4.1°S	Line 24	−3.77	−103.68	−3.90	−103.74	22VI-L14	2011/07/21 11:10	2011/07/22 00:45
S4	3.9°S-4.9°S	Line 25	−4.15	−104.64	−4.20	−106.43	22VI-L15	2011/07/22 04:50	2011/07/22 14:35
		Line 26	−4.17	−104.55	−4.31	−106.34	22VI-L16	2011/07/22 14:55	2011/07/23 04:30

**Table 2:** Calculation of effective survey lengths.

Seg	Organized Name	MAPR track length(km)		ORP track length(km)		Segment length (km)	%*
		Original	Subtract overlap	Original	Subtract overlap		
S1	Line 01	32.05	32.05	32.05	32.05	84.4	37.97%
S2–1	Line 02	11.18	0.00	11.18	0.00	47.3	88.38%
	Line 03	10.75	10.75	10.75	10.75		
	Line 04	19.88	19.88	19.88	19.88		
S2–2	–	–	–	–	–	223.9	0
S2–3	Line 05	1.53	1.53	1.53	1.53	28.6	38.37%
	Line 06	9.44	9.44	9.44	9.44		
S2–4	Line 07	13.58	13.58	13.58	13.58	151.4	47.10%
	Line 08	5.44	0.00	5.44	0.00		
	Line 09	7.61	7.61	7.61	7.61		
	Line 10	6.21	6.21	6.21	6.21		
	Line 11	4.31	4.31	4.31	4.31		
	Line 12	8.69	8.69	8.69	8.69		
	Line 13	2.88	2.88	2.88	2.88		
	Line 14	22.60	22.60	22.60	22.60		
S2–5	Line 15	15.05	15.05	15.05	15.05	127	82.65%
	Line 16	33.20	33.20	0.00	0.00		
	Line 17	14.17	14.17	0.00	0.00		
	Line 18	3.41	0.00	3.41	0.00		
	Line 19	10.23	0.00	10.23	10.23		
	Line 20	14.87	14.87	14.87	14.87		
	Line 21	3.64	0.00	0.00	0.00		
	Line 22	6.71	0.00	0.00	0.00		
	Line 23	3.69	0.00	0.00	0.00		
S3	Line 24	15.38	15.38	15.38	15.38	27.79	55.33%
S4	Line 25	12.13	12.13	12.13	12.13	67.96	42.58%
	Line 26	16.81	16.81	0.00	0.00		
**Total**		305.42	261.14	227.21	207.19	758.35	22.33%

*The percentage of segment covered by the unique tow length used for Fs calculations.

## 2 Experimental Design, Materials and Methods

### 2.1 Instrument deployments

With the advent of in situ water column sensing for hydrothermal plumes, water column surveys for axial hydrothermal activity have advanced from a reliance upon discrete single-cast CTD profiles at regular spacing along axis [2] to continuous along-axis surveys [3]. Such studies can include continuous raising and lowering of an instrumented package through the water column in "tow-yo" mode as a ship proceeds along a set path across the seafloor or, when conducting co-registered geological/geophysical investigations, by attaching multiple sensors along the same cable as the deep-tow package so that the sensors are deployed at known and fixed offsets above the deep-tow package as that deep-tow package is raised and lowered to maintain a constant survey altitude over the underlying terrain [4]. In both examples, continuous strings of time-series sensor data can be used to reconstruct 2-dimensional sections of hydrothermal water column anomalies overlying the mid-ocean ridge seafloor.

In this study, all data were collected using the second approach.

### 2.2 Choice of sensors

#### 2.2.1 Optical backscatter sensors

MAPR instrument packages include a pressure sensor, a temperature sensor and a sensor detect that detects optical back-scatter all of which are recorded within the MAPR instrument at a predetermined frequency for recover aboard ship at the end of each deployment. For all surveys reported here the variable sampling rate for the MAPR instruments was set to 5 s [5]. The presence of particles in the water column (whether mineralogical, organic or microbiological in origin) can cause an increase in light-scattering and, hence, an increase in the optical back-scatter voltage which can readily be converted to Nephelometric Turbidity Unit (NTU) values. In the deep ocean, far from continental dust and riverine inputs, suspended particle loads are typically low - except for benthic boundary layers that can form close to the seafloor, especially so in thickly sedimented areas. Mid-ocean ridges, by definition, tend to be located toward the centers of ocean basins and, further, characterized by young seafloor that is characterized by little to no sediment cover. Consequently, particle-laden hydrothermal plumes which reach levels of neutral buoyancy at narrowly-defined depth horizons, 100 m or more above the seafloor, can provide clear evidence for on-axis hydrothermal venting compared to the otherwise low-NTU oceanographic background [3]. It is on this basis that NTU anomalies have routinely been used to prospect for particle plumes along mid-ocean ridges where they are assumed to be sourced from high-temperature hydrothermal venting through precipitation of Fe oxy-hydroxides and/or polymetallic sulfides [3,[6], [7]]. The approach also relies on the fact that particle enrichments that are imparted to hydrothermal plumes can be detected, readily, above background values as these plumes are dispersed many kilometers through the water column. Consequently, the MAPR instrument packages do not need to be towed directly over an active vent-site to be able to determine that there is high temperature hydrothermal venting along any given section of ridge crest. For the East Pacific Rise, the typical rise-height that has been observed for dispersing, non-buoyant hydrothermal plumes are ∼100 m above the depth of the ridge-axis seafloor where venting occurs.

#### 2.2.2 Oxidation reduction potential (ORP) sensors

ORP sensors are electrode-based and can detect when the sensor encounters waters that are out of redox equilibrium even though they cannot determine which chemical species, in any given setting, are responsible for the signal that is detected. In prior work using autonomous underwater vehicles, it has been shown that ORP sensors can detect the presence of chemically reduced species within the relatively "fresh" portions of dispersing hydrothermal plumes, out to ∼1 km away from a high temperature submarine vent-source [8], [9]. When deployed close above the seafloor (5–50 m), ORP sensors can also detect low-temperature fluid flow, both in hydrothermal settings at mid-ocean ridges [10], [11], [12], and in association with cold seeps at continental margins [13], [14], [15], [16]. In our surveys, the ORP sensor was mounted on a deep-tow package deployed ∼5 m above the seafloor.

### 2.3 Data reduction

#### 2.3.1 Optical backscatter sensor data

Optical backscatter anomalies are routinely used to detect deep-sea hydrothermal particle plumes along Mid-Ocean Ridge axes (see reviews by [3,[6], [7]]). A particularly effective method has been used to calculate the parameter ∆NTU (the NTU value in excess above the ambient water-column background voltage, where NTU represents the nominal turbidity unit – a dimensionless unit). Following that approach, the optical back-scatter data presented in this paper has been processed as follows to generate ∆NTU values. First, we filtered the data, to remove voltage "spikes" generated from interactions of the light beam with large transient particulate organic flocs (identified as single point anomalies that exhibit ±1 sd departure from an 11-point running average: [17]). Next, for each MAPR, on each deployment, we calculated the average and standard deviation of the NTU values recorded from the sensor during its descent to the seafloor between 1500 and 2000 m - the most optically clean component of the water column. The NTU values from this deep-ocean layer define the background for each MAPR survey. While average NTU values vary across the multi-year multi-deployment data-set, arising from both seasonal and inter-instrumental variability, standard deviations all fall within ±1. We have subtracted the background average NTU value from each MAPR data-set (on a deployment by deployment basis) to calculate time-series ∆NTU values for each MAPR from each deployment. We have then taken the conservative approach of defining statistically significant optical back-scatter anomalies as those in which above-background anomalies exhibit values of ∆NTU ≥ 5.

#### 2.3.2 Oxidation-Reduction potential (ORP) sensor data

Oxidation-Reduction Potential sensors can remain stable while being deployed at near constant depth to within ±1 mV for periods of several hours [8]. For this study, we conservatively define a statistically significant ORP anomaly as one that exhibits a rapid decrease of ≥ 5 mV, before beginning recovery toward its pre-anomaly voltage, and that extends for ≤1 km across the seabed.

#### 2.3.3 Calculating effective seafloor survey lengths

In total, we conducted a cumulative length of ∼305 km of deep-tow surveys between 1.9°N and 4.9°S, EPR (Table 1 – See also, Fig. 1 in Chen et al., 2020). Much of this region was surveyed by single survey-lines along axis, but some surveys included repeat tows through the same region (for example, the 11.18 km of Line 02 was conducted to the west of Lines 03 & 04 along the same length of ridge segment). Wherever this has occurred the length of any overlapping survey has been omitted when calculating the effective length of ridge crest surveyed (Table 2). Accordingly, the ∼305 km of along-track surveys conducted (Table 1) reduces to 261 km of unique sections of ridge-axis that were surveyed using optical back scatter sensors and the corresponding ridge-lengths for ORP surveys are 227 km and 207 km, respectively (Table 2).

#### 2.3.4 Combined analysis

Once the data for each sensor have been manipulated, different permutations of the combinations of anomalies observed have been used to ascribe a specific style (*H, L, U, S*) of vent-source (Table 3).

**Table 3:** The methodology for assigning different source types.

Optical	Sensor	ORP	Interpretation	Annotation
Midwater	Seafloor	Seafloor	(Chen et al., 2020)	(Figs 1-15)
√	√	√	High Temperature	H
√	√	x	High Temperature	H
√	x	√	High Temperature	H
√	x	x	High Temperature	H
x	√	√	Low Temperature	L
x	x	√	Low Temperature	L
√	√	multiple	Undifferentiated	U
√	x	multiple	Undifferentiated	U
x	√	x	Suspected	S

### 2.4 Results

#### 2.4.1 Segment 1 (EPR 1.9–1.20°N)

Segment 1 is located between the PCG Triple Junction and the PNG Triple Junction and was investigated for hydrothermal activity by a 32-km-long deep-tow Line 01. In addition to the near-bottom ORP sensor, this deployment included two MAPRs placed at 100 m and 250 m above the deep-tow package (Fig. 1). Extensive non-buoyant particle plume anomalies are observed at ∼100 m altitude along almost the entire length of the segment, punctuated by two hiatuses. Plume ‘A’ extends from 1.88°N-1.79°N and is centered close to 1.85°N. Plume depths are 2770–2900 m with no associated ORP anomalies: source type *H* (*n* = 1). Plume area 'B' extends from 1.76 −1.65°N at ∼2770–2820 m depth. Extensive near-bottom ORP anomalies were observed during this portion of the survey, all more than 1 km apart: source types *H* (*n* = 1) and *U* (*n* = 4). Continuing south, a major ORP anomaly (#6) is observed close to 1.65°N (Region C): source type *L* (*n* = 1). An additional particle plume extends from 1.64–1.60°N at water depth 2760–2850 m (Region D) with further ORP anomalies (#7) and (#8) more than 1 km apart: source types *H* (*n* = 1) and *U* (*n* = 1).

#### 2.4.2 Segment 2-1 (EPR 1.20–0.70°N)

Segment 2–1 extends from the PNG triple junction to the first OSC at 0.70°N. No particle anomalies were observed by the +250 m MAPR but the +100 m MAPR detected intense NTU anomalies (up to 30 mV) between 1.04–0.98°N and lower intensity anomalies at the same depth range (2750–2900 m) as far as 0.91°N: a cumulative length of ≥ 15 km (Fig. 2). ORP Anomalies (#1, #2) were observed to the north of the particle plume (Region E): source type *L* (*n* = 2). An ORP anomaly (#3) coincides with the northern limit of the NTU anomalies at 1.04°N and multiple further sets of ORP anomalies (#4, #5, #6,) occur at 1.02°N-1.00°N (Region F) source types *H* (*n* = 1) and *U* (*n* = 3). Isolated ORP anomalies also occur at 0.98°N (Region G): source type *L* (*n* = 1) and at 0.95°N (Region H): source type *L* (*n* = 1).

#### 2.4.3 Segment 2–3 (EPR 1.20–1.50°S)

Segment 2–3 is a short 2nd order ridge segment that includes the volcanic seamount *Niaochao* (trans: *Bird's Nest*). Line 06 passed directly over the summit of *Niaochao* from NW to SE. MAPRs at +20 m and +70 m recorded distinct particle anomalies as they were raised upward to clear the inward facing SE wall of the crater (102.45°W) and a strongest ORP anomaly was detected at the same location (Region I): source type *H* (*n* = 1) (Fig. 3).

#### 2.4.4 Segment 2–4 (EPR 1.50–2.80°S)

One set of surveys in segment 2–4 were focused at 2.00°S - 2.02°S. Line 07 (Fig. 4) identified deep NTU anomalies at 102.59–102.56°W (Region J): source type S (*n* = 1) and optical backscatter sensor anomalies in the mid-water at both 102.60°W and at 102.61 −102.62°W. Partially overlapping line 08 did not reveal a coincident ORP anomaly at Region K: source type *S* (*n* = 1) but did record a co-registered ORP anomaly at 102.62°W in Region L: source type *H* (*n* = 1) (Fig. 5).

Further south in Segment 2–4, Line 09 (Fig. 6) detected a weak ORP signal with a co-located mid-water optical back-scatter anomaly at ∼102.635°W (Region M): source type *H*. An even more pronounced set of ORP anomalies and coincident mid-water optical backscatter anomalies are also observed further east at ∼102.625°W (Region N): source type *H* (*n* ≥ 1).

Line 10 (Fig. 7), near 2.15°S, revealed a broad ORP anomaly close to 102.62°W, coincident with prominent NTU anomalies at 2600–2870 m water depth (Region O): source type *H* (*n* = 1). A second broad ORP anomaly (Region P) was observed closer to 102.63°W, with particle-anomalies in the overlying water column at +50 m, +100 m and +150m: source type *U* (*n* = 1).

At the south end of segment 2–4, Line 11 was conducted along the ridge axis from 2.265°S to 2.240°S while Lines 12 and 13 were conducted across axis to intersect Line 11, oriented WSW-ENE and WNW-ESE respectively. Three sets of ORP anomalies were observed on Line 12 (Fig. 8). One ORP anomaly was detected near ∼102.63°W in an area that also exhibited near-continuous near-bottom NTU anomalies (Region Q): source type *L* (*n* = 1). More intense ORP anomalies were observed crossing the ridge-axis at 102.64°W, coincident with intense NTU anomalies in the overlying water column (Region R): source type *H* (*n* = 1). A third, much weaker ORP anomaly accompanied by intense near-bottom particle anomalies was observed at ∼102.655°W (Region S): source type *L* (*n* = 1). Line 13 passed across the same region of the ridge axis, at a different orientation and intercepted two of the same combinations of mid-water and seafloor anomalies as Line 12 (Region R at 102.635–650°W; Region S at ∼102.655°W) (Fig. 9). At the southernmost end of segment 2–4, Line 11 recorded prominent mid-water particle anomalies along the ridge axis from 2.265°S to 2.240°S (Fig. 10). A pronounced ORP anomaly coincided with the northern limit of that NTU anomaly (Region T): source type *H* (*n* = 1).

#### 2.4.5 Segment 2–5 (EPR 2.80–4.00°S)

Segment 2–5 extends from 2.8°S to the Quebrada fracture zone and was investigated by a series of across and along-axis surveys. In the north, Line 15 passed NW-SE across the ridge axis (Fig. 11). Two sets of ORP anomalies were intercepted close to the ridge axis. Region U (∼102.51°W) was accompanied by high near bottom NTU anomalies: source type *L* (*n* = 1). More than 1 km further east, Region V was characterized by the same combination of a pronounced ORP anomaly and deep NTU anomalies close above the seafloor: source type L (*n* = 1).

Two overlapping survey lines (18, 19) were occupied along axis between 3.00°S and 3.30°S. Starting in the north (Fig. 12), an ORP anomaly was detected on Line 18 near 3.10°S together with near-bottom NTU anomalies (Region W): source type *L* (*n* = 1). The same combination of anomalies was observed at the same latitudes on Line 19 (Fig. 13) together with an additional ORP anomaly near 3.21°S that did not show corresponding near-bottom NTU anomalies (Region X): source type *L* (*n* = 1). At a similar latitude to the region X anomaly, Line 17 (with no ORP sensor) crossed the ridge axis from NNW to SSE (Fig. 14). No NTU anomalies were observed where the survey crossed the ridge axis but strong anomalies were detected close above the seafloor to the east of the ridge axis close to 3.23°S (Region X): source type *L* (*n* = 1).

At the southern end of Segment 2–5, Line 20 crossed the ridge axis near 3.6°S (Fig. 15). West of the ridge axis, a set of near-bottom turbidity anomalies were observed near 102.66°W (Region Y): source type *S* (*n* = 1). Pronounced ORP anomalies and co-registered deep-water NTU anomalies were observed just east of the ridge-axis (Region Z): source type *L* (*n* = 1).

## Declaration of Competing Interest

The authors declare that they have no known competing financial interests or personal relationships which have, or could be perceived to have, influenced the work reported in this article.
